# Development of a sandwich ELISA for recombinant feline GM-CSF and determination of its serum half-life

**DOI:** 10.3389/fvets.2026.1958789

**Published:** 2026-10-19

**Authors:** Chun Yu, Miao Wang, Li Chen, Tianliang Wang, Ya Zhao, Hetao Song, Meilin Jin

**Affiliations:** 1 College of Veterinary Medicine, Huazhong Agricultural University, Wuhan, China; 2 Hubei Jiangxia Laboratory, Wuhan, China; 3 National Key Laboratory of Agricultural Microbiology, Huazhong Agricultural University, Wuhan, China; 4 Center for Frontier Crosses in Animal Diseases and One Health, Huazhong Agricultural University, Wuhan, China

**Keywords:** double-antibody sandwich ELISA, feline, GM-CSF, half-life, recombinant protein

## Abstract

**Introduction:**

Feline granulocyte-macrophage colony-stimulating factor (GM-CSF) is a key cytokine involved in the regulation of myeloid immune responses and represents an important therapeutic target for leukopenia. However, the rapid *in vivo* clearance of native GM-CSF requires frequent administration, which limits its clinical translation. To overcome this limitation, a recombinant GM-CSF-Fc protein was constructed using Fc fusion technology. Pharmacokinetic evaluation of GM-CSF-Fc, however, is constrained by the lack of analytical approaches capable of simultaneously quantifying differently engineered forms of recombinant GM-CSF.

**Methods:**

In this study, recombinant feline GM-CSF was expressed in a CHO eukaryotic expression system and used to immunize rabbits to generate high-affinity polyclonal antibodies. Based on these antibodies, a sandwich ELISA was established.

**Results:**

The assay exhibited excellent linearity within 7.8–2000 pg/mL (R^2^ = 0.993), and its validated quantitative range was 50–2000 pg/mL, covering the expected concentration fluctuations of recombinant GM-CSF under physiological and immune-activated conditions in cats. The assay demonstrated good precision and accuracy, and no cross-reactivity with related feline cytokines (IL-3, IL-5, M-CSF, and G-CSF) was observed. Notably, the system efficiently quantified differently engineered forms of recombinant feline GM-CSF (His-tagged and Fc-fused) with comparable recovery, overcoming the limitations of conventional assays optimized for a single protein format. Pharmacokinetic analysis following subcutaneous administration in cats showed that Fc fusion substantially prolonged the apparent terminal elimination half-life of immunoreactive GM-CSF from 5.05 ± 0.91 h to 40.89 ± 3.41 h, supporting the improved pharmacokinetic profile of the Fc-fused construct.

**Discussion:**

In conclusion, this study provides a versatile and sensitive analytical platform for the preclinical evaluation of Fc-fused feline GM-CSF formulations and serves as an important methodological reference for the quantitative assessment of Fc-fused veterinary biologicals.

## 1 Introduction

Feline granulocyte-macrophage colony-stimulating factor (GM-CSF) is a key cytokine involved in the regulation of myeloid immune responses. It promotes the proliferation and differentiation of neutrophils, macrophages, and dendritic cells and plays critical roles in anti-infectious immunity and hematopoietic homeostasis, making it a promising therapeutic target for feline leukopenia (1–3). In cats, GM-CSF supports the formation of granulocyte, macrophage, and mixed colonies from bone marrow cells and promotes dendritic cell differentiation (4). Feline panleukopenia virus (FPV) and feline immunodeficiency virus (FIV) infections frequently cause severe leukopenia, and clinical studies have shown that recombinant human GM-CSF can increase leukocyte counts in some infected cats (5).

However, native GM-CSF is rapidly cleared *in vivo* and requires frequent administration, which substantially limits its clinical translation. In veterinary medicine, the long-acting feline G-CSF fusion protein APB-F1 has demonstrated that half-life extension can sustain elevated white blood cell and neutrophil counts for up to 10 days, providing proof-of-concept that half-life extension of feline hematopoietic growth factors is feasible and therapeutically beneficial. Nevertheless, no Fc-fused feline GM-CSF formulation is currently available, and no analytical method capable of quantifying differently engineered forms of feline GM-CSF has been reported, limiting its pharmacokinetic evaluation and clinical development.

To date, no commercially available feline-specific GM-CSF detection kit has been reported. Due to the substantial sequence divergence of GM-CSF among different species, heterologous cross-species detection methods often exhibit poor recognition efficiency and potential immunological cross-reactivity, making them unsuitable for accurate quantification of feline GM-CSF (6–8). In addition, traditional bioactivity assays are generally labor-intensive, insufficiently sensitive, and poorly reproducible, limiting their application for quantitative analysis of trace GM-CSF in complex biological matrices such as serum (9). In contrast, sandwich ELISA has been widely recognized for its high sensitivity, strong specificity, good reproducibility, and resistance to matrix interference, and has been extensively applied in quantitative studies of hematopoietic cytokines and therapeutic proteins (10, 11).

Accordingly, the present study aimed to express recombinant feline GM-CSF using a CHO eukaryotic expression system, generate high-affinity polyclonal antibodies by rabbit immunization, and establish a double-antibody sandwich ELISA capable of quantifying differently engineered forms of recombinant feline GM-CSF. The assay was designed to overcome the limitation of conventional ELISA, which is applicable to only a single protein format and cannot meet the requirements for pharmacokinetic evaluation of long-acting GM-CSF formulations. The assay was further applied to a pharmacokinetic study in cats to evaluate the effect of Fc fusion on the serum persistence of immunoreactive GM-CSF. This work is expected to provide a versatile and sensitive detection tool for the preclinical evaluation of Fc-fused feline GM-CSF formulations and a methodological reference for the quantitative analysis of Fc-fused veterinary proteins.

## 2 Methods

### 2.1 Cells and antibodies

CHO cells were preserved and provided by the cell bank of our institution. EMCD CHO®104 medium was purchased from Sigma-Aldrich, USA. Feline GM-CSF antibody (Cat. No. AF987) and capture antibody (Cat. No. MAB9872) were obtained from R&D Systems, USA. AF987 was used for Western blot verification of the CHO-expressed recombinant feline GM-CSF proteins, while MAB9872 served as the capture antibody in the sandwich ELISA. Streptavidin-HRP (Cat. No. abs90469) was supplied by Absin Bioscience (Shanghai, China). HRP-conjugated goat anti-rabbit IgG (H + L) secondary antibody (Cat. No. BF03008) was purchased from Bosterlong Biotechnology (Suzhou, China). The Biotinylated Rapid Labeling Kit (Cat. No. HY-KD1113) was acquired from MedChemExpress (MCE), USA.

The detection antibody used in this study was an in-house rabbit polyclonal antibody generated by immunizing rabbits with untagged recombinant feline GM-CSF, purified by Protein A affinity chromatography, and biotinylated using the above kit. The final sandwich pair consisted of the commercial capture antibody MAB9872 and the biotinylated rabbit polyclonal detection antibody.

### 2.2 Expression, purification, and identification of recombinant proteins

The coding sequences of feline GM-CSF (GenBank: AAX63391.1) and feline IgG Fc fragment (GenBank: ATI97569.1) were retrieved from GenBank. Two recombinant fusion proteins were designed: GM-CSF-His, with an HRV 3C cleavage site, a GGGGS flexible linker, a Twin-Strep-tag, and a 10 × His tag fused sequentially at the C-terminus; and GM-CSF-Fc, with an HRV 3C cleavage site, a GGGGS linker, a Twin-Strep-tag, and a feline IgG Fc fragment fused at the C-terminus. Coding sequences were optimized for CHO codon usage and cloned into pXC17.4 to generate pXC17.4-GM-CSF-His and pXC17.4-GM-CSF-Fc. Plasmids were electrotransfected into CHO cells, and recombinant proteins were expressed in fed-batch culture. GM-CSF-His and GM-CSF-Fc were purified using Ni-NTA affinity and Protein A affinity chromatography, respectively. Protein concentration was measured by BCA assay (Thermo Scientific Pierce), and purity and specificity were verified via SDS-PAGE and Western blot. Densitometric analysis was performed using ImageJ to quantify purity.

### 2.3 Rabbit polyclonal antibody generation

Healthy female New Zealand White rabbits aged 8–12 weeks (1.5–2 kg, *n* = 5) were used. Pre-immune serum was collected as a negative control. For primary immunization, 2 mg of untagged recombinant feline GM-CSF protein was emulsified with an equal volume of complete Freund’s adjuvant and injected subcutaneously at multiple sites. Boosters were administered on days 14 and 28 with the same antigen dose emulsified with incomplete Freund’s adjuvant. Blood was collected by cardiac puncture 7 days after the final booster, and antisera were obtained by centrifugation at 4 °C. Antibody titers were determined by indirect ELISA, and the antiserum with the highest titer was selected for purification and subsequent ELISA development.

Antibody titers were determined by indirect ELISA. Briefly, microplates were coated with recombinant feline GM-CSF protein. Immune sera were serially diluted from 1:100 to 1:32000 and used as the primary antibody, while HRP-conjugated goat anti-rabbit IgG was used as the secondary antibody. After color development, OD_630_ nm values were measured. A P/N ratio (OD value of positive serum/OD value of negative serum) ≥ 2.1 was considered positive, and the highest positive dilution was defined as the antibody titer. Immune sera were preliminarily purified by saturated ammonium sulfate precipitation. The precipitates were resuspended in equilibration buffer, filtered through a 0.45 μm membrane, and further purified using a Protein A affinity chromatography column (12). After removal of non-specific proteins with equilibration buffer, specific antibodies were eluted with glycine buffer (pH 3.0–3.6). Antibody purity and specificity were verified by SDS-PAGE and Western blot analysis. Purified antibodies were dialyzed overnight against PBS (pH 7.4) and adjusted to 2 mg/mL. Antibodies were incubated with biotin at a molar ratio of 1:10 for 2 h at room temperature in the dark. The reaction products were dialyzed against PBS at 4 °C for 24 h with three buffer replacements, sterilized through a 0.22 μm membrane filter, and stored at −80 °C for subsequent sandwich ELISA construction.

### 2.4 Establishment and optimization of the double-antibody sandwich ELISA for feline GM-CSF detection

A double-antibody sandwich ELISA was established for the detection of feline GM-CSF. The standard procedure included capture antibody coating, PBST washing, blocking, sample incubation, biotinylated detection antibody binding, streptavidin-HRP incubation, TMB chromogenic reaction under dark conditions, and reaction termination with 0.25% HF solution. The absorbance was measured at 630 nm, the standard reading wavelength used in our laboratory. All samples were analyzed in triplicate, and the entire experiment was independently repeated three times to ensure data reliability. To obtain the optimal detection conditions, a checkerboard titration method was applied for the systematic gradient optimization of key ELISA parameters. For antibody pairing optimization, the capture antibody was set at concentrations of 2, 4, 6, and 8 μg/mL, while the biotinylated detection antibody was diluted to 1, 1.5, 2, and 2.5 μg/mL to screen the optimal antibody combination. For blocking condition optimization, four blocking reagents, including 2% skim milk, 5% skim milk, 2% BSA, and 5% BSA, were tested at 37 °C for blocking durations of 0.5, 1, and 2 h to determine the most efficient blocking system. In addition, streptavidin-HRP was serially diluted with PBST to working ratios of 1:2000, 1:5000, 1:10000, and 1:20000 and incubated at 37 °C for 0.5, 1, and 2 h, respectively. The optimal dilution ratio and incubation duration were determined accordingly, thereby establishing an optimized ELISA system for feline GM-CSF detection.

The detailed procedure was as follows:

(1) Coating: the capture antibody was diluted to 2, 4, 6, and 8 μg/mL in coating buffer and added to 96-well plates at 100 μL/well, followed by incubation at 4 °C overnight or at 37 °C for 2 h;
(2) Washing: the coating solution was discarded, and plates were washed five times with PBST (3 min each) and patted dry;
(3) Blocking: 200 μL of 2% skim milk, 5% skim milk, 2% BSA, or 5% BSA was added to each well and incubated at 37 °C for 0.5, 1, or 2 h; the blocking solution was discarded, and plates were washed five times with PBST (3 min each) and patted dry;
(4) Sample incubation: GM-CSF standards at different concentrations and test samples were added at 100 μL/well and incubated at 37 °C for 30 min; the liquid was discarded, and plates were washed five times with PBST (3 min each) and patted dry;
(5) Detection antibody: biotinylated rabbit anti-feline GM-CSF polyclonal antibody was diluted to 1, 1.5, 2, and 2.5 μg/mL in dilution buffer and added at 100 μL/well, followed by incubation at 37 °C for 30 min; the liquid was discarded, and plates were washed five times with PBST (3 min each) and patted dry;
(6) Streptavidin-HRP: streptavidin-HRP was diluted in PBST to 1:2000, 1:5000, 1:10000, and 1:20000 and added at 100 μL/well, followed by incubation at 37 °C for 0.5, 1, or 2 h; the liquid was discarded, and plates were washed five times with PBST (3 min each) and patted dry;
(7) Color development: 100 μL of freshly prepared TMB substrate was added to each well and incubated at 37 °C in the dark for 15 min;
(8) Termination and measurement: 50 μL of 0.25% HF solution was added to each well to stop the reaction, and absorbance was measured at 630 nm using a microplate reader.

### 2.5 Standard curve and assay validation

#### 2.5.1 Standard curve

The feline GM-CSF-His standard was serially diluted at a two-fold ratio to prepare twelve concentration gradients of 16,000, 8,000, 4,000, 2000, 1,000, 500, 250, 125, 62.5, 31.3, 15.6, and 7.8 pg/mL, with 0 pg/mL as a blank control. A standard curve was fitted and plotted using a four-parameter logistic regression model with a weighting factor of 1/y^2^, with the standard concentration as the abscissa (X-axis) and the average OD value as the ordinate (Y-axis), and the corresponding regression equation was established. Subsequently, the obtained regression equation was applied for back-calculation analysis of test samples. The quantitative range was defined as 50–2000 pg/mL, and the 4PL model was used for quantification of all PK samples.

#### 2.5.2 Limit of detection and limit of quantification

Fifty negative serum samples collected from healthy cats were tested to determine the limit of detection (LOD) and calculated limit of quantification (calculated LOQ) of the established method. The LOD and calculated LOQ were calculated based on the mean and standard deviation (SD) of the OD values of negative samples, following the classical formulas (13): LOD = Mean + 3 × SD and calculated LOQ = Mean + 10 × SD.

The experimentally validated lower limit of quantification (LLOQ) was defined as the lowest QC concentration meeting both precision (%CV ≤ 20%) and accuracy (%RE within ±20%). Calibrators below the validated LLOQ were used only for standard curve fitting and were not included in the quantitative range.

#### 2.5.3 Precision and accuracy

Furthermore, six gradient concentrations of recombinant feline GM-CSF quality control samples (50, 100, 200, 400, 800, and 1,600 pg/mL) were selected to verify the precision and accuracy of the developed ELISA method. Independent tests were performed in five separate batches on different days. The repeatability and accuracy of the method were evaluated by calculating the coefficient of variation (%CV), relative bias (%bias), and total error (TE). The calculation formulas were as follows (14): %CV = (SD/Mean) × 100%, %bias = (measured concentration − theoretical concentration)/theoretical concentration × 100%, and TE = |%CV| + |%bias|.

#### 2.5.4 Specificity of the feline GM-CSF sandwich ELISA

Feline IL-3, IL-5, M-CSF, and G-CSF were used as cross-reactive proteins and diluted to 10, 100, 1,000, and 2000 pg/mL in sample diluent. Recombinant feline GM-CSF was used as the positive control, and sample diluent as the negative control. All samples were measured using the established sandwich ELISA, and the OD₆₃₀ value of each well was recorded. The P/N ratio was calculated as the OD value of the cross-reactive sample (P) divided by the OD value of the negative control (N). A P/N ratio > 2.1 was considered indicative of cross-reactivity, whereas a P/N ratio ≤ 2.1 indicated no cross-reactivity.

#### 2.5.5 Quantitative equivalence between GM-CSF-His and GM-CSF-Fc

To evaluate whether GM-CSF-His and GM-CSF-Fc produce comparable quantitative responses, concentration-response curves were generated for both proteins under identical assay conditions. GM-CSF-Fc was serially two-fold diluted using the same concentration gradient as the GM-CSF-His standard (16,000, 8,000, 4,000, 2000, 1,000, 500, 250, 125, 62.5, 31.3, 15.6, and 7.8 pg/mL, with 0 pg/mL as the blank control). Both proteins were analyzed on the same microplate in triplicate, and the experiment was independently repeated three times. Data were fitted using a four-parameter logistic (4PL) regression model, and the EC50, Hill slope, and maximum signal (Top) of the two curves were compared. The EC50 ratio (GM-CSF-Fc/GM-CSF-His) and its 95% confidence interval were calculated. Statistical comparisons of EC50 and Hill slope between the two curves were performed using the extra sum-of-squares F test in GraphPad Prism 8.0, with *p* > 0.05 indicating no significant difference.

#### 2.5.6 Dilution integrity and parallelism, and serum spike recovery

Blank feline serum (endogenous GM-CSF < LLOQ) was spiked with GM-CSF-His or GM-CSF-Fc at 10,000 pg/mL and serially diluted 1:5, 1:10, 1:20, 1:40, and 1:80 with blank feline serum. Each dilution was prepared in triplicate and analyzed in three independent runs. Concentrations were calculated from the standard curve and multiplied by the dilution factor to obtain back-calculated concentrations.

The same spiked serum samples were used to evaluate dilution integrity, parallelism, and serum spike recovery. Recovery (%) was calculated as (back-calculated concentration/spiked concentration) × 100%. Parallelism was assessed by comparing the CV of back-calculated concentrations across different dilution factors. Acceptance criteria were recovery 80–120%, %RE within ± 20%, and CV ≤ 20%.

### 2.6 Determination of serum half-life in cats

A total of 6 healthy adult cats aged 8–12 months with a body weight of 2.5–3.5 kg were randomly assigned into two experimental groups (*n* = 3 per group). All cats were neutered females. All cats were confirmed healthy by physical examination, negative PCR results for common feline infectious pathogens, and normal hematological and biochemical parameters. Cats were randomly assigned using a random number table, and no animals were excluded from the analysis.

After one week of adaptive rearing, the cats were subcutaneously injected with 10 μg/kg recombinant feline GM-CSF-His or GM-CSF-Fc protein, designated as the GM-CSF-His and GM-CSF-Fc groups, respectively. Peripheral blood samples were collected from the cephalic vein of the forelimb at 0, 1, 2, 4, 8, 16, 24, 32, 48, 72, 96, 120, 144, 168, 192, and 216 h post-administration. The collected blood samples were allowed to clot at 4 °C, followed by centrifugation at 3000 rpm for 5 min to isolate serum, which was stored at −20 °C. Samples exceeding the ULOQ (2000 pg/mL) were diluted with blank feline serum into 50–2000 pg/mL using dilution factors of 1:5–1:80; dilution integrity and parallelism are described in Section 2.5.6.

Serum GM-CSF concentrations were quantified using the validated sandwich ELISA. Concentrations below the LLOQ (50 pg/mL) were reported as BLQ; absorption-phase BLQ values were set to zero, and elimination-phase BLQ values were excluded from the terminal phase regression, which included at least three time points ≥ LLOQ. Pharmacokinetic parameters, including the terminal elimination half-life, were calculated using DAS 2.0 software (BioGuider, China) by non-compartmental analysis.

### 2.7 Ethics and consent to participate

The Animal Care and Use Committee of Huazhong Agriculture University approved all the animal experimental procedures (Approval No. HZAURAB-2026-0009) and adhered strictly to the Institutional Animal Care Use Protocols. This study was conducted in accordance with the ARRIVE guidelines.

### 2.8 Statistical analysis

All data were analyzed using SPSS 22.0 software (IBM, USA). All results were expressed as the mean ± standard deviation (SD). Variance homogeneity was assessed by the F-test. When variances were unequal, Welch’s t-test was applied; otherwise, an independent-samples t-test was used. Differences were considered significant at *p* < 0.05 and highly significant *p* < 0.01. Given the small sample size (*n* = 3 per group), the statistical comparison should be interpreted with caution, and the interpretation primarily relies on individual pharmacokinetic profiles and descriptive differences between groups.

## 3 Result

### 3.1 Expression and identification of recombinant proteins

To obtain recombinant feline GM-CSF and its Fc fusion proteins (GM-CSF-His and GM-CSF-Fc), the coding sequence of feline GM-CSF was retrieved from GenBank, and the amino acid sequences of the two recombinant proteins were designed accordingly (Figure 1A). Following affinity purification, SDS-PAGE analysis showed that the molecular weights of GM-CSF-His and GM-CSF-Fc were approximately 25 kDa and 55 kDa, respectively, which were consistent with their theoretical values. Western Blot analysis confirmed that both proteins could be specifically recognized by anti-feline GM-CSF antibodies (Figures 1B,C). Grayscale quantification of Western Blot bands revealed that the purities of GM-CSF-His and GM-CSF-Fc reached 84.23 and 93.40%, respectively (Figure 1D). The protein concentrations determined by the BCA assay were 1,621 μg/mL and 1919 μg/mL (Figure 1E). Collectively, these results demonstrate that the eukaryotic CHO expression system enables stable production of recombinant feline GM-CSF proteins with high purity, providing high-quality immunogens and calibration standards for subsequent antibody preparation and ELISA establishment.

**Figure 1:**
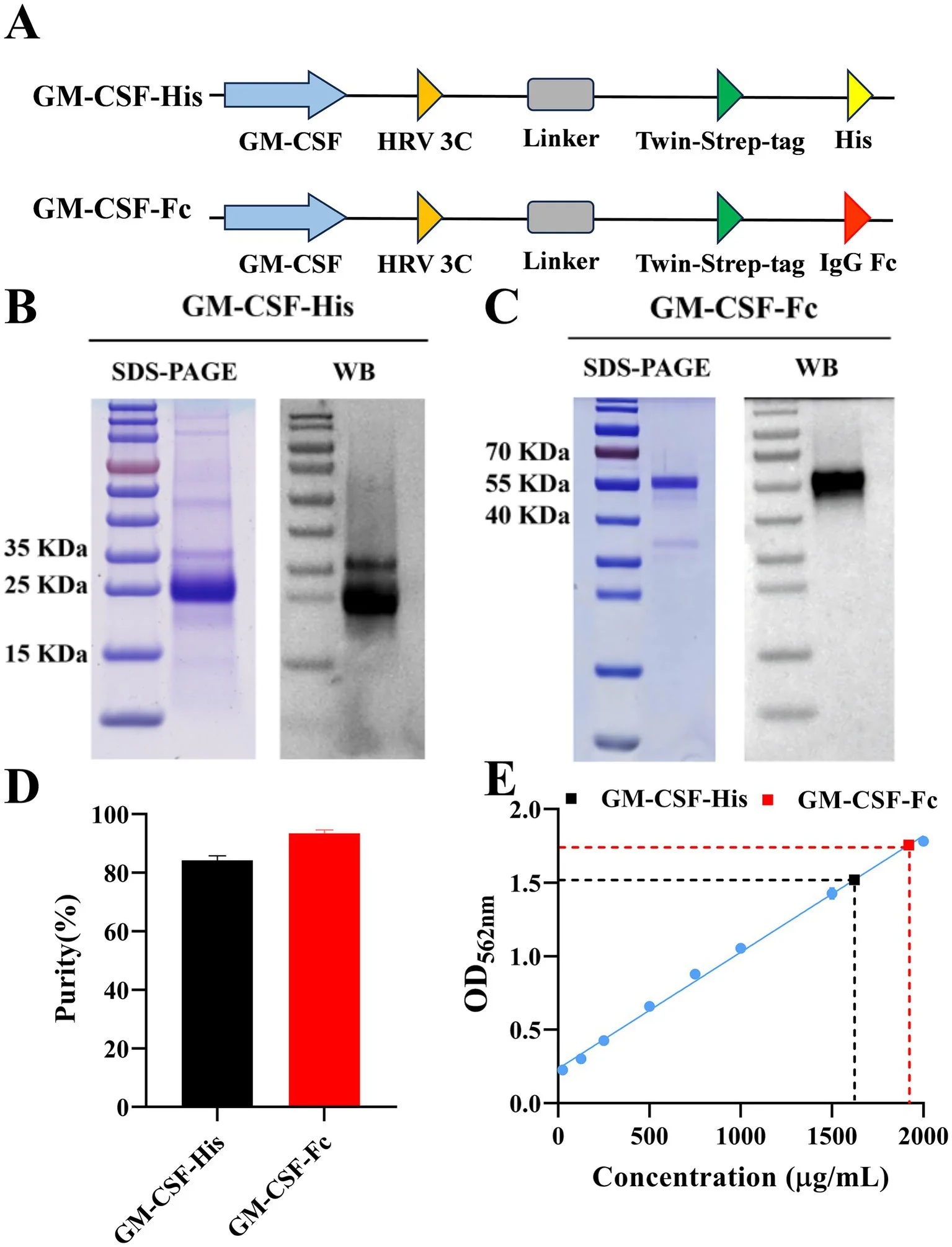
Expression and identification of recombinant feline GM-CSF-His and GM-CSF-Fc proteins. **(A)** Schematic diagram of the amino acid sequence structure of recombinant feline GM-CSF-His and GM-CSF-Fc fusion proteins. **(B,C)** SDS-PAGE and Western blot detection results of purified recombinant feline GM-CSF-His and GM-CSF-Fc. **(D)** Grayscale quantitative analysis of electrophoretic bands for GM-CSF-His and GM-CSF-Fc proteins. **(E)** The concentrations of purified GM-CSF-His and GM-CSF-Fc were quantified via the BCA method. The standard curve equation was fitted as Y = 0.0007920X + 0.2351 with a coefficient of determination R^2^ = 0.996.

### 3.2 Preparation of polyclonal antibodies

Recombinant feline GM-CSF protein was used as the immunogen for rabbit immunization. Serum samples were collected after primary and booster immunizations, and polyclonal antibodies were purified sequentially via saturated ammonium sulfate precipitation and Protein A affinity chromatography. SDS-PAGE analysis showed distinct protein bands corresponding to the IgG heavy chain (approximately 50 kDa) and light chain (approximately 25 kDa) with negligible impurity bands, indicating favorable antibody purity (Figure 2A). Western Blot verification confirmed that the prepared polyclonal antibody could specifically bind to recombinant feline GM-CSF-His and GM-CSF-Fc antigen (Figures 2B,C). The concentration of the purified antibody was determined to be 5 mg/mL by the BCA assay.

**Figure 2:**
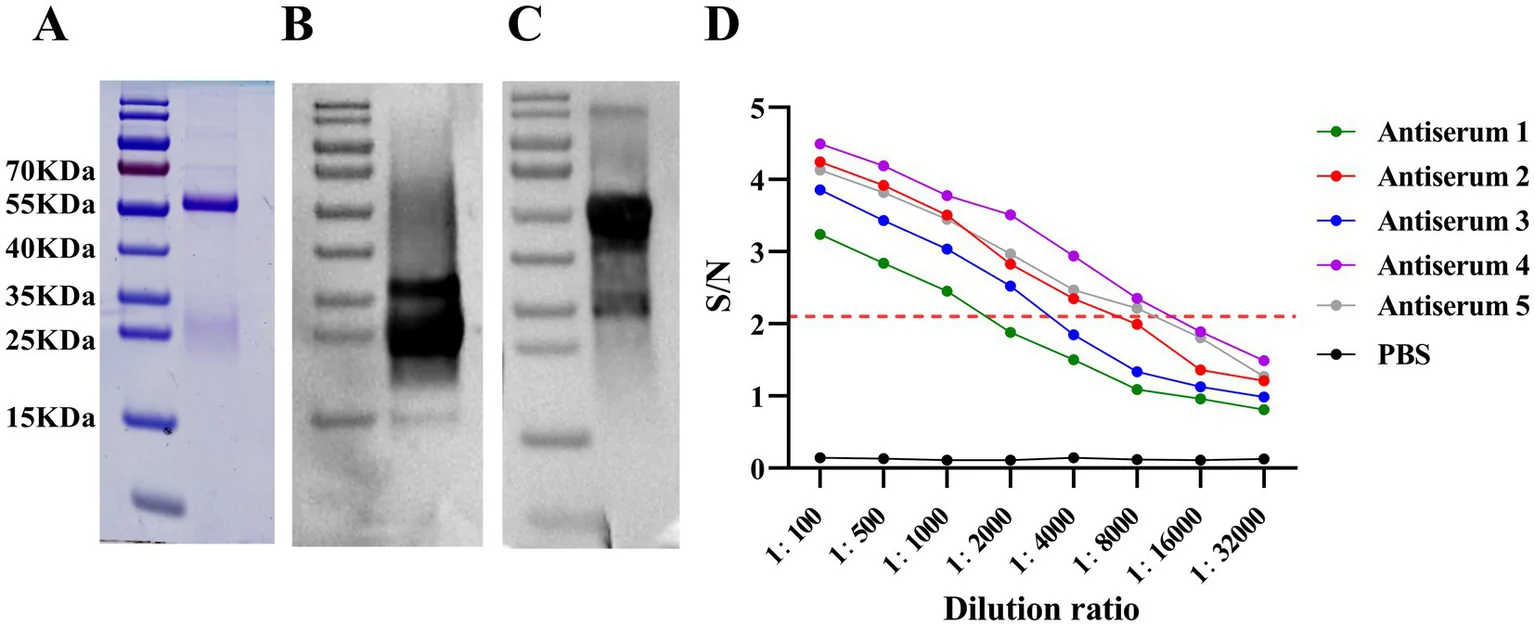
Titer and specificity analysis of rabbit anti-feline GM-CSF polyclonal antibodies. **(A)** SDS-PAGE analysis of rabbit polyclonal antibodies after purification from antiserum. **(B,C)** Western blot analysis verifying the specific binding of the purified polyclonal antibodies to recombinant GM-CSF-His and GM-CSF-Fc proteins, respectively. **(D)** Antibody titer results of rabbit polyclonal antibodies detected by indirect ELISA. The abscissa represents the serial dilution ratio of immunized rabbit serum, and the ordinate represents the signal-to-noise (S/N) ratio of absorbance between samples and blank controls. The red horizontal line indicates the positive threshold (S/N = 2.1); values above the line were defined as antibody-positive, while those below are antibody-negative.

Indirect ELISA was performed to detect the antibody titer of immunized serum. The serum samples were serially two-fold diluted from 1:100 to 1:32,000, and PBS was set as the blank control to calculate the signal-to-noise (S/N) ratio. The results showed that the S/N ratios of immune serum gradually decreased with increasing dilution ratios, indicating a stable overall immune response. Using an S/N ratio > 2.1 as the positive criterion, the titer of Antiserum 1 was 1:1,000; the titer of Antiserum 3 was 1:2,000; the titer of Antiserum 2 was 1:4,000; and the titers of Antiserum 4 and Antiserum 5 reached 1:8,000. Among all samples, Antiserum 4 exhibited the highest S/N ratio at each dilution gradient and showed the optimal overall titer and specificity. Notably, the high-titer serum (Antiserum 4) still maintained an S/N ratio above the positive threshold even at a dilution of 1:8,000 (Figure 2D). These findings confirmed that the immunization protocol applied in this study could induce robust and stable specific antibody responses. The qualified polyclonal antibodies with high immune reactivity and favorable specificity provide reliable raw materials for the subsequent construction of the ELISA detection system.

### 3.3 Establishment and optimization of the double-antibody sandwich ELISA for feline GM-CSF

Checkerboard titration was performed to optimize the working concentrations of capture and detection antibodies for the established double-antibody sandwich ELISA system. Recombinant feline GM-CSF standard at 1000 pg/mL was used as the positive sample, and the standard dilution buffer was set as the negative control to screen the optimal antibody pairing combination (Table 1). The results showed that the positive sample yielded an OD value of 1.225 and the highest P/N ratio of 5.083 when the capture antibody concentration was 6 μg/mL and the detection antibody concentration was 2 μg/mL (Figure 3A). This combination exhibited a significantly higher P/N value than other concentration groups. Therefore, 6 μg/mL for the capture antibody and 2 μg/mL for the detection antibody were determined as the optimal working concentrations for the developed sandwich ELISA.

**Table 1:** Optimization of capture and detection antibody concentrations for feline GM-CSF double-antibody sandwich ELISA.

Capture antibody (μg/mL)	Detection antibody (μg/mL)											
	1			1.5			2			2.5		
	P	N	P/N	P	N	P/N	P	N	P/N	P	N	P/N
2	0.723	0.245	2.955	0.869	0.232	3.751	1.043	0.256	4.072	1.012	0.263	3.843
4	0.865	0.257	3.365	1.017	0.215	4.735	1.186	0.265	4.481	0.997	0.295	3.855
6	1.010	0.231	4.381	1.075	0.237	4.528	1.225	0.241	5.083	0.903	0.238	3.785
8	0.835	0.222	3.756	0.961	0.217	4.427	0.974	0.244	3.986	0.746	0.235	3.454

P stands for the absorbance value measured from GM-CSF positive samples; N corresponds to the absorbance value of blank negative controls; P/N refers to the signal-to-noise ratio of the sandwich ELISA system. The optimal antibody pair was selected based on the maximum P/N value.

**Figure 3:**
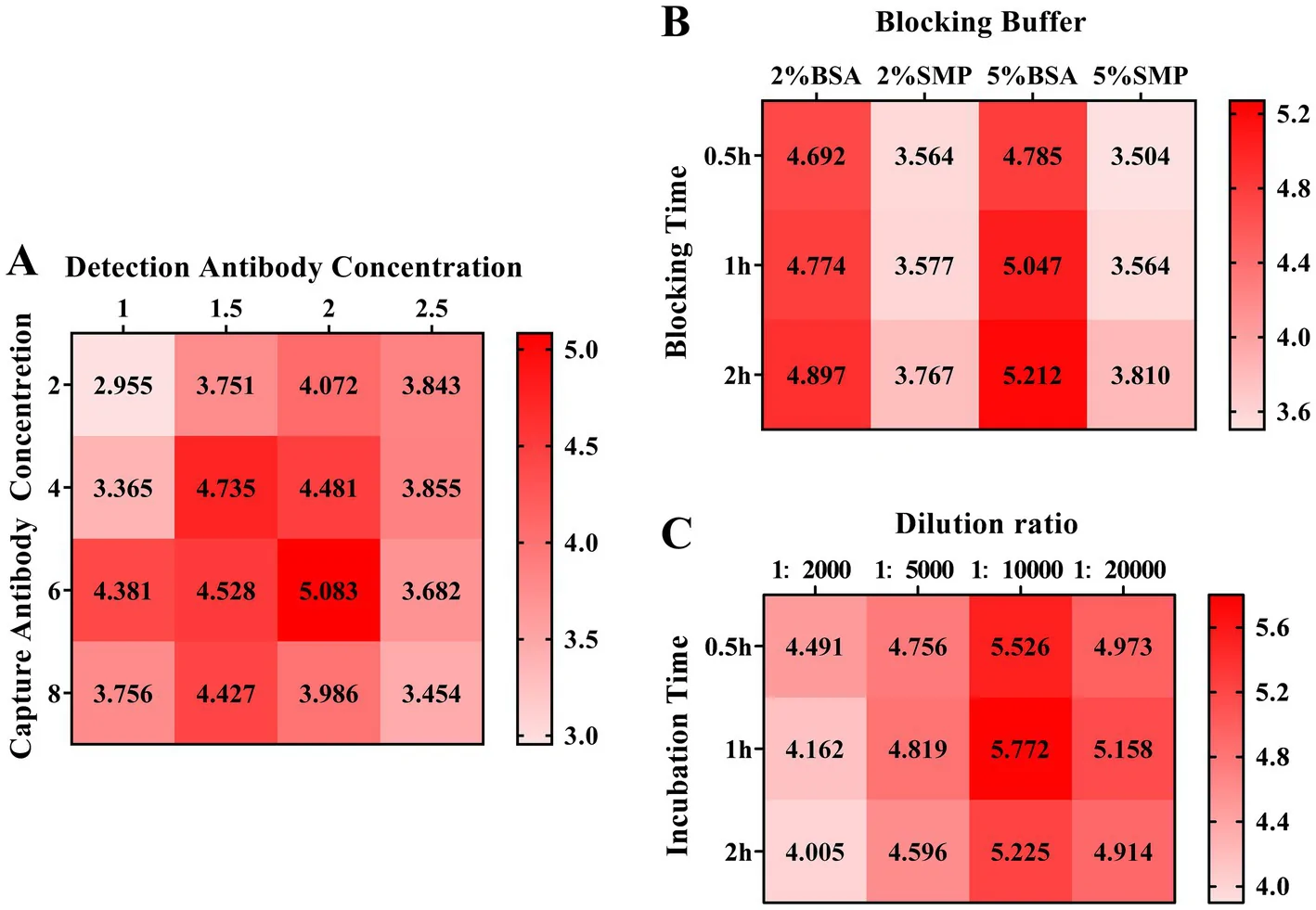
Optimization of antibody pairing, blocking conditions and streptavidin-HRP working parameters for feline GM-CSF sandwich ELISA. **(A)** Matrix screening for optimal working concentrations of capture and detection antibodies; the x-axis represents detection antibody concentration and the y-axis represents capture antibody concentration. **(B)** Optimization of blocking reagent and incubation time; the x-axis indicates blocking reagent type and the y-axis denotes blocking duration. **(C)** Optimization of streptavidin-HRP dilution ratio and incubation time; the x-axis shows dilution ratio and the y-axis stands for incubation period.

On the basis of the optimized antibody pairing conditions, the blocking parameters of the ELISA system were further screened via checkerboard titration (Table 2). Four blocking reagents, including 2% BSA, 5% BSA, 2% skim milk powder, and 5% skim milk powder, were applied with gradient incubation durations to explore the optimal blocking condition. The results demonstrated that blocking with 5% BSA for 2 h achieved the optimal detection performance, with a low negative OD value of 0.191 and a peak P/N ratio of 5.212 (Figure 3B). This condition effectively reduced non-specific binding and background signals of the detection system. Consequently, blocking with 5% BSA for 2 h was confirmed as the optimal blocking condition for the present ELISA system.

**Table 2:** Optimization of blocking conditions for feline GM-CSF double-antibody sandwich ELISA.

Blocking time	Blocking reagent											
	2% BSA			2% skim milk powder			5% BSA			5% skim milk powder		
	P	N	P/N	P	N	P/N	P	N	P/N	P	N	P/N
0.5 h	1.232	0.263	4.692	1.019	0.286	3.564	1.061	0.222	4.785	0.969	0.277	3.504
1 h	1.147	0.240	4.774	0.959	0.268	3.577	1.050	0.208	5.047	0.910	0.255	3.564
2 h	1.129	0.231	4.897	0.921	0.244	3.767	0.995	0.191	5.212	0.896	0.235	3.810

P stands for the absorbance value measured from GM-CSF positive samples; N corresponds to the absorbance value of blank negative controls; P/N refers to the signal-to-noise ratio of the sandwich ELISA system. The optimal blocking condition was selected based on the maximum P/N value.

After determining the optimal antibody pairing and blocking conditions, the working dilution and incubation duration of streptavidin-HRP were further optimized (Table 3). Four dilution gradients (1:2000, 1:5000, 1:10000, and 1:20000) combined with gradient incubation times were evaluated to screen the optimal reaction parameters. The results showed that a 1:10000 dilution of streptavidin-HRP with 1 h incubation yielded the optimal detection performance, with a positive OD value of 1.315, a low negative background OD value of 0.228, and the highest P/N ratio of 5.772 among all groups (Figure 3C). This condition effectively maintained a strong positive detection signal while significantly reducing background interference. Therefore, a dilution of 1:10000 and an incubation time of 1 h were defined as the optimal working conditions for streptavidin-HRP in the established sandwich ELISA system.

**Table 3:** Optimization of streptavidin-HRP working conditions for feline GM-CSF double-antibody sandwich ELISA.

Incubation time	Streptavidin-HRP dilution											
	1:2000			1:5000			1:10000			1:20000		
	P	N	P/N	P	N	P/N	P	N	P/N	P	N	P/N
0.5 h	1.777	0.396	4.491	1.618	0.340	4.756	1.194	0.216	5.526	1.015	0.204	4.973
1 h	1.856	0.446	4.162	1.716	0.356	4.819	1.315	0.228	5.772	1.098	0.213	5.158
2 h	1.935	0.483	4.005	1.846	0.402	4.596	1.388	0.266	5.225	1.169	0.238	4.914

P stands for the absorbance value measured from GM-CSF positive samples; N corresponds to the absorbance value of blank negative controls; P/N refers to the signal-to-noise ratio of the sandwich ELISA system. The optimal streptavidin-HRP dilution and incubation time were selected based on the maximum P/N value.

### 3.4 Standard curve establishment and methodological validation

#### 3.4.1 Standard curve and quantitative range

To achieve accurate quantification of feline GM-CSF in samples, a total of 12 serial concentrations of recombinant feline GM-CSF standards ranging from 7.8 to 16,000 pg/mL were prepared under the optimized ELISA reaction system, with 0 pg/mL as a blank control. The OD₆₃₀ nm values and corresponding standard concentrations were fitted using a four-parameter logistic (4PL) regression model with a weighting factor of 1/y^2^ in GraphPad Prism. The full-range standard curve exhibited a coefficient of determination (R^2^) of 0.995 (Figure 4A). Linear regression analysis of the 7.8–2000 pg/mL segment gave an R^2^ value of 0.993 (Figure 4B), verifying reliable linearity for precise quantification within this concentration interval.

**Figure 4:**
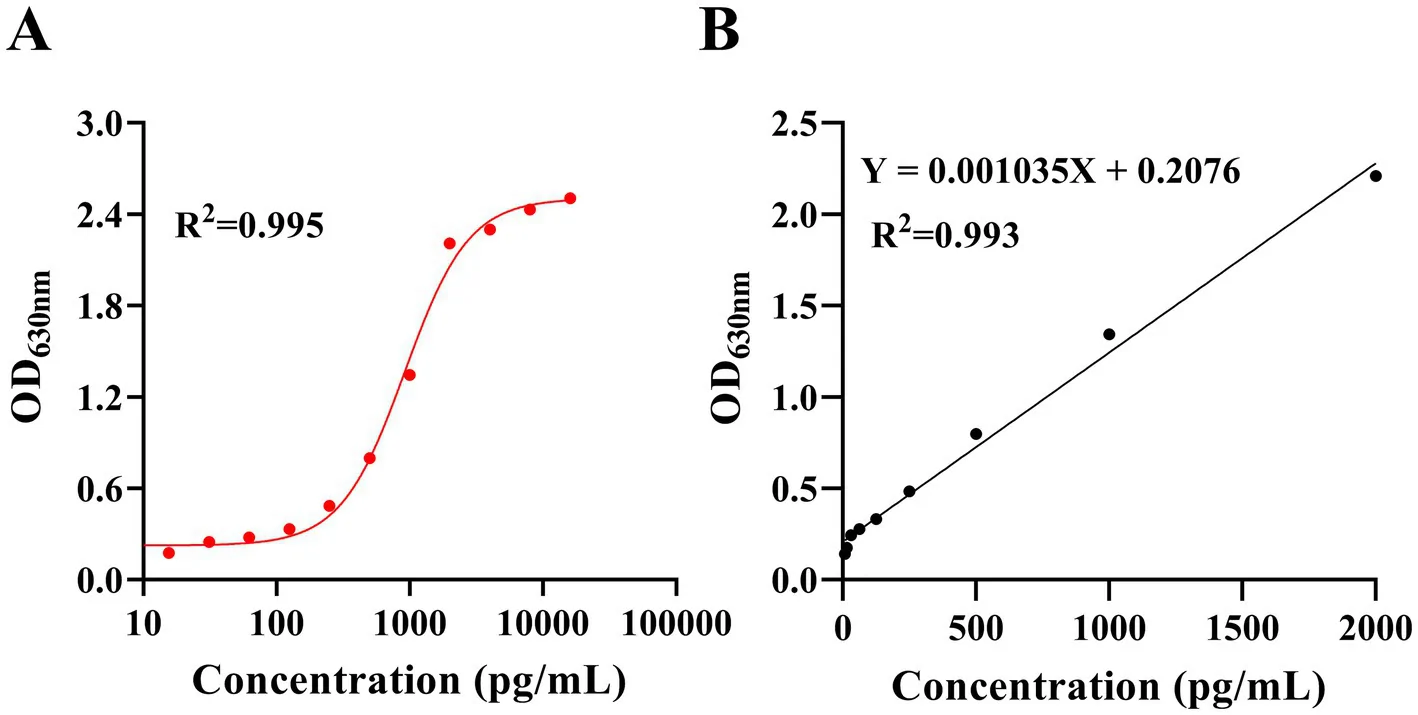
Standard curve and linear detection range of the established double-antibody sandwich ELISA for recombinant feline GM-CSF. **(A)** Full-range standard curve fitted by four-parameter logistic regression. The OD₆₃₀ _nm_ signal exhibited a typical S-shaped dose–response relationship with standard concentrations, with a high fit (R^2^ = 0.995). **(B)** Magnified view of the linear range of 7.8–2000 pg/mL. A strong linear correlation between OD values and GM-CSF concentrations was observed in this interval. The regression equation was Y = 0.001035X + 0.2076, with R^2^ = 0.993, which is reliable for the accurate quantification of test samples.

Furthermore, back-calculation analysis was performed for each concentration point in the linear range, and the methodological performance was evaluated based on the coefficient of variation (%CV) and relative bias (%bias). The acceptance criteria were a deviation within ±20% of the nominal concentration (±25% at the LLOQ), with at least 75% of calibrators meeting this criterion. The two lowest concentrations (7.8 and 15.6 pg/mL) were excluded from the quantitative range due to bias exceeding 20%, and all remaining calibrators met the criteria. The final validated quantitative range was 50–2000 pg/mL (Table 4).

**Table 4:** Standard curve back-calculation verification of feline GM-CSF ELISA.

Replicate	Parameter	Theoretical concentration (pg/mL)						
		31.3	62.5	125	250	500	1,000	2000
1	Measured conc	35.69	64.20	119.09	241.15	569.53	1093.42	1911.49
	%CV	1.63%	2.00%	1.45%	6.51%	1.98%	1.49%	2.99%
	%bias	14.21%	2.72%	−4.73%	−3.54%	13.91%	9.34%	−4.43%
2	Measured conc	31.15	66.04	129.63	288.89	550.98	1073.13	1925.40
	%CV	3.30%	4.89%	3.08%	3.03%	3.00%	0.92%	0.23%
	%bias	−0.33%	5.66%	3.70%	14.56%	10.20%	7.31%	−3.73%
3	Measured conc	33.18	63.91	118.90	276.31	566.90	1108.98	1920.48
	%CV	4.82%	4.65%	2.91%	2.39%	1.61%	1.73%	1.55%
	%bias	6.17%	2.26%	−4.88%	10.53%	13.38%	10.90%	−3.98%
4	Measured conc	32.21	64.59	114.07	248.98	575.62	1145.22	1868.87
	%CV	4.36%	4.90%	3.92%	8.28%	2.22%	3.29%	2.79%
	%bias	3.08%	3.34%	−8.75%	−0.41%	14.12%	14.52%	−6.56%
5	Measured conc	35.11	70.29	117.64	283.48	572.14	1068.68	2024.08
	%CV	1.69%	3.57%	4.03%	4.62%	3.99%	1.86%	3.69%
	%bias	12.35%	12.46%	−5.89%	13.39%	14.43%	6.87%	1.20%

Measured conc refers to sample concentrations calculated from the standard curve. %CV stands for coefficient of variation reflecting assay precision, while %bias means relative deviation reflecting assay accuracy. Calibrators at 31.3 pg/mL and below were used for standard curve fitting; however, after the validated LLOQ was determined to be 50 pg/mL, concentrations below 50 pg/mL were not included in the final quantitative range.

#### 3.4.2 Limit of detection and limit of quantification

The limit of detection (LOD) and limit of quantification (LOQ) of the established ELISA assay were determined using negative serum samples collected from healthy cats. The OD₆₃₀ nm values of fifty negative feline serum samples were measured, yielding a mean OD value of 0.207 and a standard deviation of 0.0029. Using the formulas LOD = mean + 3 × SD and calculated LOQ = mean + 10 × SD, the critical OD values for LOD and LOQ were determined to be 0.216 and 0.236, respectively. These critical OD values were then interpolated into the standard curve to obtain the corresponding concentrations. The calculated LOQ was 27.44 pg/mL, and the LOD was 8.12 pg/mL. The experimentally validated lower limit of quantification (LLOQ), defined as the lowest QC concentration meeting both precision (%CV ≤ 20%) and accuracy (%RE within ±20%), was 50 pg/mL. Calibrators at 15.6 and 31.3 pg/mL were used only for standard curve fitting and were not included in the quantitative range. The final validated quantitative range was 50–2000 pg/mL. Collectively, these data demonstrate that the developed ELISA exhibits good sensitivity for low-concentration sample detection.

#### 3.4.3 Precision and accuracy

To systematically evaluate the practical detection performance and stability of the established ELISA system, six concentrations of quality control samples (50, 100, 200, 400, 800, and 1,600 pg/mL) were set up for batch repeated detection (Table 5). Methodological validation data showed that the coefficient of variation (%CV) of each sample ranged from 0.94 to 8.31%, the relative bias (%bias) ranged from −5.12 to 12.18%, and the total error (TE) varied from 3.49 to 16.97%. The overall intra-assay precision (total %CV), average bias, and average total error were 1.79% ~ 5.83%, −3.85% ~ 10.14%, and 4.86% ~ 13.20%, respectively. All indicators met the standard requirements for quantitative detection of biological samples (Table 5). In summary, the %CV, %bias, and TE values of all quality control samples were within the commonly accepted criteria for bioanalytical method validation. These results indicate that the developed ELISA method possesses favorable precision and accuracy, which is suitable for the stable and reliable quantitative analysis of GM-CSF in feline serum samples.

**Table 5:** Validation of recombinant feline GM-CSF sandwich ELISA.

Replicate	Parameter	Theoretical concentration (pg/mL)					
		50	100	200	400	800	1,600
1	Measured concentration	56.09	111.55	208.19	418.87	776.44	1561.17
	%CV	1.56%	5.42%	5.18%	6.82%	2.11%	1.43%
	%bias	12.18%	11.55%	4.10%	4.72%	−2.94%	−2.43%
	TE (%)	13.74%	16.97%	9.28%	11.54%	5.05%	3.86%
2	Measured concentration	53.19	101.89	194.66	442.06	771.61	1559.23
	%CV	3.52%	3.74%	8.31%	2.47%	7.34%	0.94%
	%bias	6.38%	1.89%	−2.67%	10.52%	−3.55%	−2.55%
	TE (%)	9.90%	5.63%	10.98%	12.98%	10.89%	3.49%
3	Measured concentration	52.22	103.82	217.86	441.10	759.05	1550.54
	%CV	1.13%	1.67%	7.94%	4.61%	1.43%	1.81%
	%bias	4.45%	3.82%	8.93%	10.27%	−5.12%	−3.09%
	TE (%)	5.58%	5.49%	16.87%	14.88%	6.55%	4.90%
4	Measured concentration	55.50	101.89	218.82	396.64	767.74	1546.67
	%CV	4.14%	1.69%	2.22%	4.27%	3.40%	3.27%
	%bias	11.00%	1.89%	9.41%	−0.84%	−4.03%	−3.33%
	TE (%)	15.15%	3.58%	11.63%	5.11%	7.43%	6.60%
5	Measured concentration	55.50	106.72	194.66	380.21	760.98	1536.04
	%CV	2.99%	0.97%	7.60%	3.62%	7.76%	1.50%
	%bias	11.00%	6.72%	−2.67%	−4.95%	−4.88%	−4.00%
	TE (%)	14.00%	7.69%	10.27%	8.56%	12.63%	5.50%
Total	Measured concentration	55.07	105.51	204.09	409.45	769.19	1550.78
	%CV	3.06%	2.96%	5.83%	4.29%	5.15%	1.79%
	%bias	10.14%	5.51%	2.04%	2.36%	−3.85%	−3.08%
	TE (%)	13.20%	8.47%	10.54%	9.55%	9.00%	4.86%

Measured conc refers to sample concentrations calculated from the standard curve. %CV stands for coefficient of variation reflecting assay precision, while %bias means relative deviation reflecting assay accuracy. TE (%) = |%CV| + |%bias|. The lowest QC concentration (50 pg/mL) corresponds to the validated LLOQ. The “Total” row represents inter-assay precision and accuracy across five independent batches. Acceptance criteria: %CV ≤ 15%, %bias within ±15%, and TE ≤ 20%.

#### 3.4.4 Specificity of the feline GM-CSF sandwich ELISA

The specificity of the established ELISA was evaluated using feline IL-3, IL-5, M-CSF, and G-CSF as cross-reactive proteins at 10, 100, 1,000, and 2000 pg/mL. Recombinant feline GM-CSF was used as the positive control, and sample diluent as the negative control. The P/N ratio was calculated as the OD value of the cross-reactive sample divided by that of the negative control, with P/N > 2.1 indicating cross-reactivity.

The P/N ratios of all four cytokines at all tested concentrations were below 2.1, indicating no cross-reactivity. The positive control GM-CSF showed P/N ratios above 2.1 at 100, 1000, and 2000 pg/mL, whereas at 10 pg/mL the P/N ratio was below 2.1, consistent with the LLOQ of 50 pg/mL (Figure 5A). These results demonstrate that the established ELISA specifically recognizes feline GM-CSF and shows no significant cross-reactivity with feline IL-3, IL-5, M-CSF, or G-CSF.

**Figure 5:**
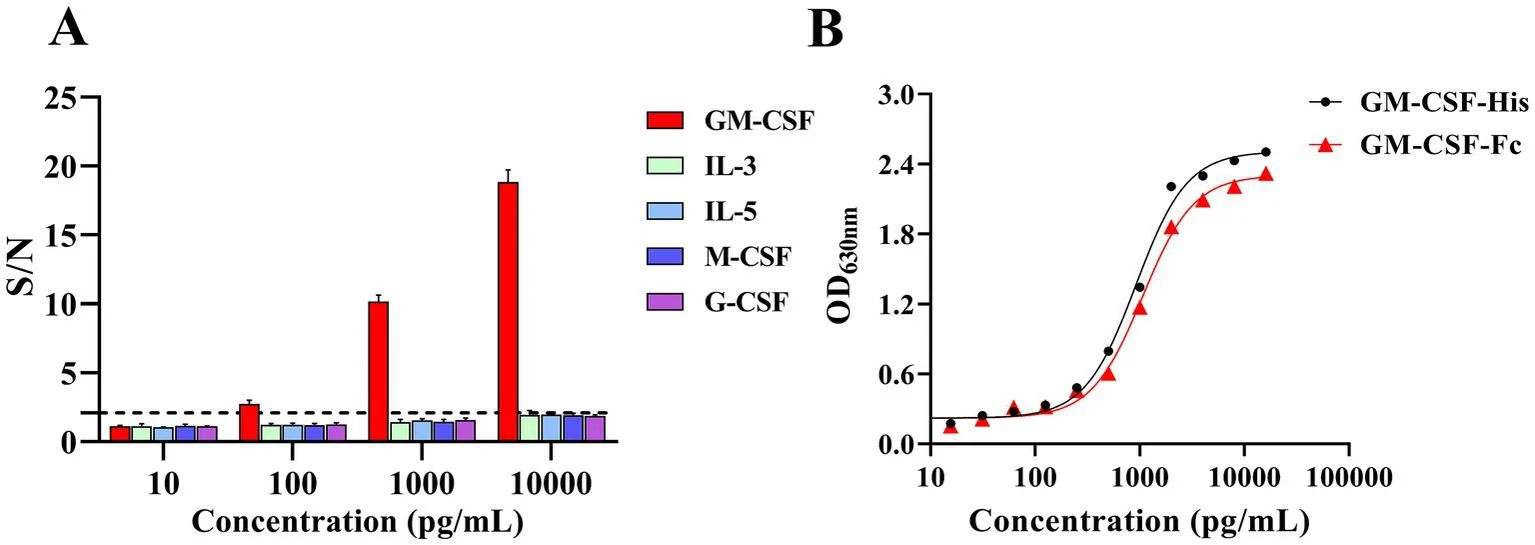
Specificity and quantitative equivalence of the established sandwich ELISA. **(A)** Cross-reactivity of the established ELISA with related feline cytokines. Feline IL-3, IL-5, M-CSF, and G-CSF were tested at 10, 100, 1,000, and 2000 pg/mL, with recombinant feline GM-CSF as the positive control. The dashed line indicates the S/N threshold of 2.1. **(B)** Concentration-response curves of GM-CSF-His and GM-CSF-Fc generated under identical assay conditions. Both proteins produced typical sigmoidal curves, with EC50 values of 918.0 pg/mL (GM-CSF-His) and 1,064 pg/mL (GM-CSF-Fc), giving an EC50 ratio of 1.16 (*p* = 0.1729). Hill slopes were 1.817 and 1.729, respectively (*p* = 0.7959). Data are shown as mean ± SD from three independent experiments (*n* = 3).

#### 3.4.5 Quantitative equivalence between GM-CSF-His and GM-CSF-Fc

To evaluate whether GM-CSF-His and GM-CSF-Fc produce comparable quantitative responses, concentration-response curves were generated under identical assay conditions. Both proteins produced typical sigmoidal curves (Figure 5B). The EC50 values were 918.0 pg/mL for GM-CSF-His and 1,064 pg/mL for GM-CSF-Fc, giving an EC50 ratio (Fc/His) of 1.16. Statistical comparison using the extra sum-of-squares F test showed no significant difference in EC50 (*F* (1, 16) = 2.036, *p* = 0.1729), and the preferred model shared a single EC50 for both datasets. The Hill slopes were 1.817 and 1.729, respectively, with no significant difference (*F* (1, 16) = 0.0692, *p* = 0.7959) and a preferred model sharing a common slope. The maximum signals differed by less than 10%, indicating highly comparable and parallel curves. These results demonstrate that GM-CSF-His and GM-CSF-Fc have comparable concentration-response curves in buffer.

#### 3.4.6 Dilution integrity, parallelism, and serum spike recovery

The spiked serum samples diluted 1:5–1:80 yielded recoveries of 98.05–110.83% for GM-CSF-His and 84.86–90.35% for GM-CSF-Fc, with CVs ≤ 2.76% and bias within ±15.14% (Table 6). All values met the acceptance criteria (recovery 80–120%, CV ≤ 20%, bias ±20%). The CVs of back-calculated original concentrations across different dilutions were ≤ 20% for both proteins, supporting parallelism with the standard curve.

**Table 6:** Dilution integrity of GM-CSF-His and GM-CSF-Fc.

Group	Dilution factor	Nominal concentration (pg/mL)	Measured concentration (pg/mL)	Back-calculated concentration (pg/mL)	Recovery (%)	%CV	%bias
GM-CSF-His	1:80	125	138.53 ± 6.55	11082.68 ± 523.91	110.83 ± 5.24	2.76 ± 0.93	10.83 ± 5.24
	1:40	250	251.29 ± 5.68	10051.49 ± 227.34	100.51 ± 2.27	2.42 ± 0.92	0.51 ± 2.27
	1:20	500	509.95 ± 3.23	10198.94 ± 64.66	101.99 ± 0.65	1.48 ± 0.68	1.99 ± 0.65
	1:10	1,000	980.49 ± 9.82	9804.88 ± 98.20	98.05 ± 0.98	1.13 ± 0.14	−1.95 ± 0.98
	1:5	2000	2176.31 ± 89.80	10881.56 ± 448.98	108.82 ± 4.49	0.94 ± 0.15	8.82 ± 4.49
GM-CSF-Fc	1:80	125	107.06 ± 3.75	8564.72 ± 299.88	85.65 ± 300	2.13 ± 1.20	−14.35 ± 3.00
	1:40	250	221.87 ± 3.98	8874.91 ± 159.14	88.75 ± 1.59	2.16 ± 0.87	−11.25 ± 1.59
	1:20	500	451.74 ± 4.65	9034.73 ± 93.03	90.35 ± 0.93	1.67 ± 1.14	−9.65 ± 0.93
	1:10	1,000	848.55 ± 16.27	8485.52 ± 162.69	84.86 ± 1.63	1.11 ± 0.48	−15.14 ± 1.63
	1:5	2000	1718.95 ± 34.98	8594.73 ± 174.88	85.95 ± 1.75	1.6 ± 0.47	−14.05 ± 1.75

Nominal concentration = spiked concentration / dilution factor (theoretical concentration after dilution). Measured concentration = concentration directly read from the standard curve. Back-calculated concentration = measured concentration × dilution factor. Recovery (%) = (measured concentration / nominal concentration) × 100% = (back-calculated concentration/spiked concentration) × 100%. CV (%) = (SD / mean) × 100%. Bias (%) = [(measured concentration − nominal concentration) / nominal concentration] × 100%. Acceptance criteria: recovery 80–120%, CV ≤ 20%, bias within ±20%. Spiked concentration: 10,000 pg/mL.

Since the same spiked serum samples were used, these results simultaneously demonstrate dilution integrity, parallelism, and serum spike recovery, confirming that GM-CSF-His and GM-CSF-Fc can be accurately quantified in feline serum using the same calibration curve.

### 3.5 Pharmacokinetics of GM-CSF-His and GM-CSF-Fc in cats

To evaluate the effect of Fc fusion modification on prolonging the *in vivo* half-life of recombinant feline GM-CSF, the validated double-antibody sandwich ELISA system established in this study was applied to detect the dynamic serum concentrations of GM-CSF-His and GM-CSF-Fc after subcutaneous administration in cats (*n* = 3 per group). Serum samples with concentrations exceeding the ULOQ (2000 pg/mL) were diluted with blank feline serum at validated dilution factors (1:5–1:80) before analysis. Concentrations below the LLOQ (50 pg/mL) were reported as BLQ. The mean serum concentration-time curves were plotted accordingly (Figure 6). Following subcutaneous administration, both proteins showed an initial rise to peak concentration followed by a decline. The early decline likely reflects combined absorption, distribution, and elimination processes, which cannot be definitively separated without intravenous administration data. The terminal phase was used to estimate the apparent terminal elimination half-life (t₁/₂z).

**Figure 6:**
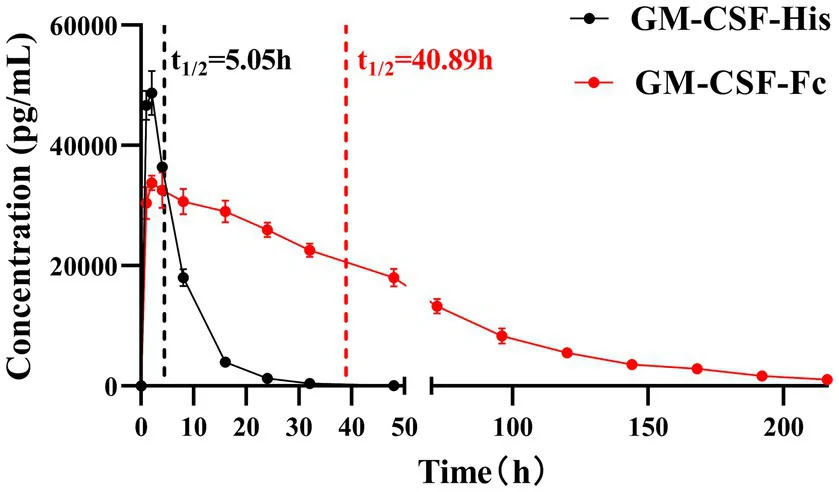
*In vivo* serum concentration-time curves of feline GM-CSF-His and GM-CSF-Fc after a single subcutaneous administration (10 μg/kg). Data are shown as mean ± SD (*n* = 3 cats per group at each time point). The black dashed line indicates the apparent terminal elimination half-life of GM-CSF-His (5.05 ± 0.91 h), and the red dashed line indicates that of GM-CSF-Fc (40.89 ± 3.41 h). Concentrations below the LLOQ (50 pg/mL) were reported as BLQ and excluded from the terminal phase regression.

Non-compartmental analysis performed with DAS 2.0 software revealed that the elimination half-lives of GM-CSF-His and GM-CSF-Fc were 5.05 ± 0.91 h and 40.89 ± 3.41 h, corresponding to an approximately 8.1-fold increase (Table 7). The mean difference was 35.84 h (95% CI: 28.04–43.65 h; t = 17.59; df = 2.284; *p* = 0.0017). Individual half-life values were clearly separated between the two groups, with no overlap (GM-CSF-His: 6.077, 4.345, and 4.724 h; GM-CSF-Fc: 39.717, 38.224, and 44.735 h). In the GM-CSF-His group, concentrations at 72 h and later were below the LLOQ and were excluded from the terminal-phase regression; λz and t₁/₂z were determined by DAS 2.0 software using its automatic terminal-phase selection. In the GM-CSF-Fc group, all concentrations remained above the LLOQ throughout the sampling period.

**Table 7:** Pharmacokinetic parameters of GM-CSF-His and GM-CSF-Fc after subcutaneous administration in cats.

Parameter	Unit	GM-CSF-His (*n* = 3)	GM-CSF-Fc (*n* = 3)	P
T_max	h	1.67 ± 0.58	2.67 ± 1.15	0.2739
C_max	pg/mL	49124.11 ± 3095.94	34050.67 ± 1768.74	0.0045
t₁/₂z	h	5.05 ± 0.91	40.89 ± 3.41	0.0017
AUC₀₋ₜ	pg/mL·h	384675.03 ± 4778.89	2293491.67 ± 178918.30	0.0029
AUC₀₋∞	pg/mL·h	385048.80 ± 4945.35	2358152.34 ± 198545.55	0.0033
MRT₀₋∞	h	6.42 ± 0.13	62.32 ± 2.40	0.0006
CLz/F	mL/h/kg	51.95 ± 0.67	8.52 ± 0.73	< 0.0001
Vz/F	mL/kg	378.31 ± 68.43	500.60 ± 15.46	0.0801

Data are presented as mean ± SD (*n* = 3 per group). Dose: 10 μg/kg. Statistical comparison between groups was performed using Welch’s t-test. Differences were considered significant at *p* < 0.05 and highly significant at *p* < 0.01.t. Tmax, time to maximum concentration; Cmax, maximum serum concentration; t₁/₂z, apparent terminal elimination half-life; AUC, area under the concentration-time curve; MRT, mean residence time; CLz/F, apparent clearance; Vz/F, apparent volume of distribution.

These findings confirmed that Fc fusion modification could significantly prolong the apparent serum persistence of immunoreactive GM-CSF in cats. Furthermore, the established ELISA accurately quantified recombinant feline GM-CSF in both His-tagged and Fc-fused formats, with comparable recovery, as confirmed by concentration-response curve comparison, serum spike recovery, and parallelism experiments. This validated assay is suitable for the pharmacokinetic evaluation of differently modified recombinant feline GM-CSF products and provides a reliable detection tool for pharmacodynamic and pharmacokinetic studies of Fc-fused recombinant cytokine preparations.

## 4 Discussion

Feline granulocyte-macrophage colony-stimulating factor (GM-CSF) is a core cytokine that modulates myeloid immune responses and serves as a crucial therapeutic target for leukopenia. Nevertheless, native GM-CSF exhibits rapid *in vivo* metabolic clearance, necessitating frequent administration and severely restricting its clinical application (5, 15). To address this limitation, this study successfully constructed a recombinant GM-CSF-Fc protein using Fc fusion technology and the CHO eukaryotic expression system, which is widely used for recombinant glycoprotein production and can support mammalian-type glycosylation and proper protein folding. Accurate quantification of trace levels of serum GM-CSF in different engineered forms is an essential technical prerequisite for pharmacokinetic evaluation and translational research. Currently, no commercial detection kit is specifically available for feline GM-CSF. Moreover, existing detection methods are typically optimized for a single protein format and cannot quantify both His-tagged and Fc-fused GM-CSF variants, which greatly hinders the systematic evaluation of Fc-fused GM-CSF preparations. Therefore, there is an urgent need to establish a sensitive and broadly applicable quantitative detection system for feline GM-CSF.

The specificity and affinity of antibodies primarily depend on the conformational integrity of antigens. Prokaryotic expression systems lack the capacity for glycosylation and correct protein folding, which easily leads to the loss of conformational epitopes and poor antibody quality (16). In contrast, the CHO eukaryotic expression system is widely used for recombinant glycoprotein production and is generally capable of performing mammalian-type glycosylation and supporting proper protein folding. However, direct experimental evidence for the glycosylation status and native conformation of the recombinant proteins produced in this study was not obtained (17, 18). Previous studies have confirmed that glycosylation significantly enhances the immune-inducing activity of cytokines. Glycosylated interferons and GM-CSF, among others, can induce specific antibodies with higher titers and superior affinity (19–21). In this study, recombinant feline GM-CSF-His and GM-CSF-Fc proteins were expressed using the CHO eukaryotic expression system, and highly specific polyclonal antibodies with a titer up to 1:8000 were successfully obtained via rabbit immunization, providing a reliable foundation for the subsequent establishment of the ELISA detection system.

Double-antibody sandwich ELISA is a classic technique for the quantitative detection of low-abundance cytokines, characterized by good specificity, sensitivity, and repeatability. It has been widely applied for the precise quantification of trace cytokines such as IL-10 and IFN-γ in serum samples (22–25). Compared with indirect and competitive ELISA, sandwich ELISA relies on dual-epitope recognition to effectively reduce serum matrix interference, making it particularly suitable for detecting trace targets in complex biological samples (22, 25). In this study, key reaction parameters including antibody concentrations, blocking conditions, streptavidin-HRP dilution ratio, and incubation duration were systematically optimized via checkerboard titration. The optimal reaction conditions were determined as follows: 6 μg/mL capture antibody, 2 μg/mL detection antibody, 2 h blocking with 5% BSA, and 1:10000 dilution of streptavidin-HRP. Methodological validation showed that the assay exhibited good linearity within 7.8–2000 pg/mL (R^2^ = 0.993), with a calculated LOQ of 27.44 pg/mL and an experimentally validated LLOQ of 50 pg/mL. Calibrators at 7.8 and 15.6 pg/mL were excluded from the quantitative range due to bias exceeding 20%, and the final validated quantitative range was 50–2000 pg/mL. The precision (%CV < 10%), accuracy (%bias < 15%), and total error (TE < 20%) all complied with the guidelines for biological sample analysis. In addition, dilution integrity, parallelism, serum spike recovery, and cross-reactivity with related feline cytokines were evaluated, and no cross-reactivity was observed (cross-reactivity < 0.1%). Notably, the standard curve was constructed using PBS-based calibrators to objectively evaluate antibody binding characteristics, whereas LOD and LOQ were determined using negative serum from healthy cats to simulate real biological matrix conditions. However, matrix effects, freeze–thaw stability, short- and long-term stability, robustness, and high-dose hook effect were not comprehensively evaluated, and these are acknowledged as limitations. The core innovation of this study lies in the ability of the established ELISA method to quantify differently engineered forms of recombinant feline GM-CSF, namely His-tagged GM-CSF-His and Fc-fused GM-CSF-Fc, thereby overcoming the limitations of conventional assays that are optimized for a single protein format. Traditional ELISA assays are generally designed for a single tagged protein, requiring independent method development for each modified variant. Such separate detection strategies not only increase experimental costs and systematic errors but also prevent parallel comparative analysis (26). Fc fusion is a well-established strategy for prolonging the half-life of cytokines by enhancing *in vivo* retention through the FcRn recycling mechanism (27). Using the assay developed herein, which recognizes the GM-CSF moiety irrespective of C-terminal modification, methodological discrepancies were minimized, enabling accurate characterization of the pharmacokinetic differences between the two recombinant proteins. Evidence for comparable quantitative recovery of the two forms was provided by concentration-response curve comparison, serum spike recovery, and parallelism experiments.

Pharmacokinetic analysis after subcutaneous administration in cats revealed distinct metabolic profiles. The half-life of GM-CSF-His was only 5.05 ± 0.91 h, indicating rapid *in vivo* clearance and a need for frequent administration. In contrast, the half-life of GM-CSF-Fc was significantly extended to 40.89 ± 3.41 h, representing an approximately 8.1-fold extension, which is consistent with the improved metabolic properties of Fc-modified feline GM-CSF (28). This 8.1-fold extension is at the higher end of the range reported for long-acting veterinary cytokines. For comparison, an Fc-fused feline interferon-ω2 (FeIFN-ω2-Fc) extended the plasma half-life from 5.80 ± 1.75 h to 34.05 ± 6.36 h (approximately 5.9-fold), with further extension to 40.55 ± 6.61 h (7.0-fold) after introducing YTE mutations (28) Similarly, the long-acting feline G-CSF fusion protein APB-F1 extended the half-life approximately 5-fold (13.3 h vs. 2.7 h) compared with native feline G-CSF (29). Notably, although the native half-lives of feline IFN-ω2 (5.80 h) and GM-CSF (5.05 h) are comparable, Fc fusion extended IFN-ω2 by only ~5.9-fold, whereas GM-CSF-Fc achieved an 8.1-fold extension. This difference may be attributed to distinct receptor-mediated clearance rates, the impact of the Fc fusion site on receptor binding, and differences in glycosylation patterns. These results support the conclusion that Fc fusion prolongs the apparent serum persistence of immunoreactive GM-CSF. However, an elimination half-life alone is not sufficient to establish a clinical dosing interval, and determining an appropriate dosing schedule would require pharmacodynamic, efficacy, and safety data.

Nevertheless, several limitations of the present study should be acknowledged. First, methodological validation was primarily performed using recombinant proteins, and the detection performance for endogenous native GM-CSF under pathological conditions remains to be verified. Second, only IL-3, IL-5, M-CSF, and G-CSF were tested for cross-reactivity; comprehensive screening against all possible feline cytokines was not performed, and the broad applicability of this method needs to be addressed in future studies. Third, matrix effects, freeze–thaw stability, short- and long-term stability, robustness, and high-dose hook effect were not comprehensively evaluated. Fourth, pharmacokinetic experiments were conducted only in healthy cats; further investigations using clinical leukopenia models are required to fully evaluate the practical application of this detection system in disease scenarios. Fifth, the sample size (*n* = 3 per group) is very small, which limits statistical power; therefore, the statistical comparison should be interpreted with caution, and the conclusions are primarily based on individual pharmacokinetic profiles and descriptive differences between the two groups rather than on statistical significance alone. Sixth, although the recombinant proteins were expressed using a CHO eukaryotic expression system, direct experimental evidence for complete glycosylation and native conformation was not obtained in this study; future studies could include glycosidase treatment, lectin blotting, or biophysical analyses to confirm these properties. Finally, this study measured only immunoreactive GM-CSF and did not assess its biological activity; therefore, the observed prolongation of serum persistence should not be equated with prolonged biological activity. In this study, a double-antibody sandwich ELISA was successfully established for the quantification of differently engineered forms of recombinant feline GM-CSF (His-tagged and Fc-fused). This assay achieves high sensitivity, good linearity (R^2^ = 0.993), and satisfactory precision and accuracy, overcoming the technical drawbacks of conventional assays optimized for a single protein format. *In vivo* pharmacokinetic analysis using this method confirmed that Fc fusion significantly prolonged the apparent terminal elimination half-life of immunoreactive GM-CSF from 5.05 h to 40.89 h (an approximately 8.1-fold extension), providing direct *in vivo* pharmacokinetic evidence that Fc fusion prolongs the apparent serum persistence of immunoreactive GM-CSF.

The ELISA platform developed herein, which recognizes the GM-CSF moiety irrespective of C-terminal modification, enables accurate quantification of differently modified recombinant feline GM-CSF and serves as a valuable methodological reference for pharmacokinetic assessment and novel biopharmaceutical development of Fc-fusion veterinary cytokines.

## 5 Conclusion

In this study, recombinant feline GM-CSF proteins were successfully expressed using the CHO eukaryotic expression system. Using these purified recombinant proteins as immunogens, specific rabbit-derived polyclonal antibodies were generated. Based on the optimized antibody pair, a double-antibody sandwich ELISA method was established for the quantitative detection of recombinant feline GM-CSF. Systematic optimization and methodological validation demonstrated that the developed ELISA system exhibits high sensitivity, with a limit of detection (LOD) of 8.12 pg/mL and a calculated LOQ of 27.44 pg/mL, and an experimentally validated LLOQ of 50 pg/mL (final quantitative range: 50–2000 pg/mL). This assay accurately recognizes and quantifies differently engineered forms of recombinant feline GM-CSF (His-tagged and Fc-fused), demonstrating good precision and accuracy and no cross-reactivity with related feline cytokines (Figure 7).

**Figure 7:**
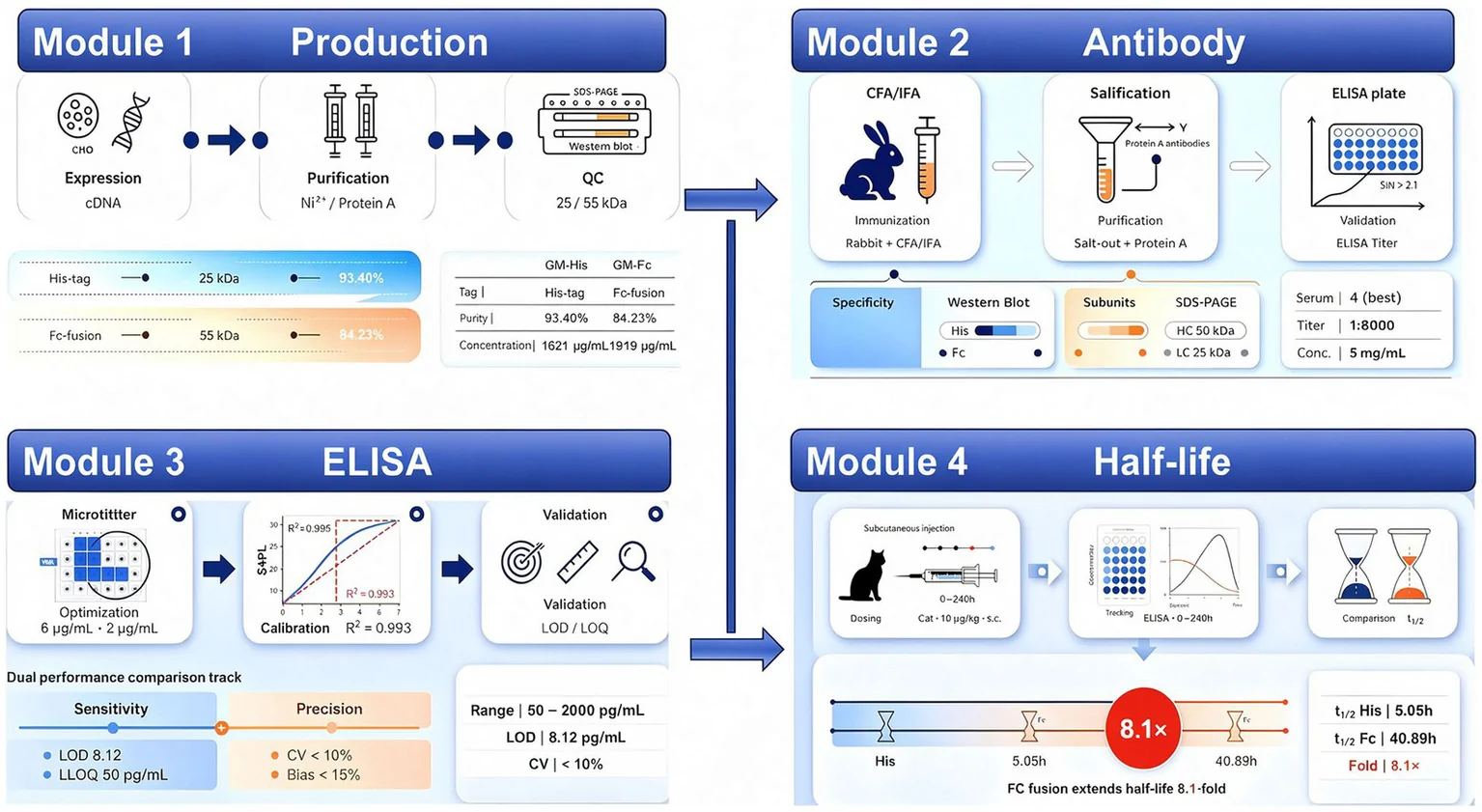
Research workflow for the development and application of the feline GM-CSF sandwich ELISA. The workflow includes four modules: (1) production of recombinant GM-CSF-His and GM-CSF-Fc in CHO cells; (2) generation and purification of rabbit polyclonal antibodies (*n* = 5 rabbits); (3) establishment and validation of the sandwich ELISA (LOD 8.12 pg/mL; LLOQ 50 pg/mL; quantitative range 50–2000 pg/mL); and (4) pharmacokinetic analysis in cats (n = 3 per group) after subcutaneous administration of 10 μg/kg GM-CSF-His or GM-CSF-Fc, showing an 8.1-fold extension of the apparent terminal elimination half-life (5.05 h vs. 40.89 h).

Collectively, the established ELISA method enables reliable and precise detection of CHO-expressed recombinant feline GM-CSF in complex serum matrices with good sensitivity and broad applicability. With minor parameter adjustments, this platform can be applied to sample detection throughout the entire process of recombinant protein expression and purification, providing a robust technical foundation for optimizing GM-CSF preparation procedures and supporting subsequent *in vivo* pharmacokinetic and functional studies.

## Data Availability

The original contributions presented in the study are included in the article/Supplementary material, further inquiries can be directed to the corresponding authors.

## References

[1] Burgess AW Camakaris J Metcalf D. Purification and properties of colony-stimulating factor from mouse lung-conditioned medium. J Biol Chem. (1977) 252:1998–2003. doi: 10.1016/S0021-9258(18)71855-3, 300377

[2] Chen P Zhang W Cui Y Sun M Dong X Li W . Porcine GM-CSF and APS as a novel complex immunostimulant improves the immune effect of pseudorabies inactivated vaccine. Vet Microbiol. (2025) 304:110453. doi: 10.1016/j.vetmic.2025.110453, 40054056

[3] Shi Y Liu CH Roberts AI Das J Xu G Ren G . Granulocyte-macrophage colony-stimulating factor (GM-CSF) and T-cell responses: what we do and don’t know. Cell Res. (2006) 16:126–33. doi: 10.1038/sj.cr.7310017, 16474424

[4] Dunham SP Bruce J. Isolation, expression and bioactivity of feline granulocyte-macrophage colony-stimulating factor. Gene. (2004) 332:97–106. doi: 10.1016/j.gene.2004.02.023, 15145059

[5] Arai M Darman J Lewis A Yamamoto JK. The use of human hematopoietic growth factors (rhGM-CSF and rhEPO) as a supportive therapy for FIV-infected cats. Vet ImmunolImmunopathol. (2000) 77:71–92. doi: 10.1016/s0165-2427(00)00232-4, 11068067

[6] Kwak S-S Cheong S-A Jeon Y Hyun S-H. Porcine granulocyte-macrophage colony-stimulating factor improves the in vitro development of cloned pocine embryos. J Vet Med Sci. (2012) 74:1095–102. doi: 10.1292/jvms.12-0050 22531103

[7] Sparrow LG Metcalf D Hunkapiller MW Hood LE Burgess AW. Purification and partial amino acid sequence of asialo murine granulocyte-macrophage colony stimulatingfactor. Proc Natl Acad Sci USA. (1985) 82:292–6. doi: 10.1073/pnas.82.2.292, 3871523 PMC397023

[8] Wong GG Witek JS Temple PA Wilkens KM Leary AC Luxenberg DP . Human GM-CSF: molecular cloning of the complementary DNA and purification of the natural and recombinant proteins. Science. (1985) 228:810–5. doi: 10.1126/science.3923623, 3923623

[9] Thorpe R Wadhwa M Bird CR Mire-Sluis AR. Detection and measurement of cytokines. Blood Rev. (1992) 6:133–48. doi: 10.1016/0268-960x(92)90025-l, 1422282

[10] Aydin S Emre E Ugur K Aydin MA Sahin İ Cinar V . An overview of ELISA: a review and update on best laboratory practices for quantifying peptides and proteins in biological fluids. J IntMed Res. (2025) 53:3000605251315913. doi: 10.1177/03000605251315913, 39922798 PMC11808753

[11] Nirmala C Trivedi R Prasanthi PV Mullangi R Srinivas NR. Development and validation of a sensitive ELISA for quantitation of Grastim (rhG-CSF) in rat plasma: application to a pharmacokinetic study. Biomed Chromatogr. (2006) 20:945–50. doi: 10.1002/bmc.625, 16506263

[12] Munir A Anjum AA Altaf I Awan AR. Immuno-affinity chromatography for purification of IgG from hyper-immune sera raised against 146S fraction of foot and mouth disease virus for diagnostic purposes. Pol J Vet Sci. (2023) 26:393–3. doi: 10.24425/pjvs.2023.145045, 37727100

[13] Armbruster DA Pry T. Limit of blank, limit of detection and limit of quantitation. Clin Biochem Rev. (2008) 29:S49–52. PMC2556583 18852857

[14] Qiu J Yang Y Kong J Cao Y Liu Y Luo H . Quantification of pharmacokinetic profiles of a recombinant canine PD-1 fusion protein by validated sandwich ELISA method. Front Vet Sci. (2022) 9:951176. doi: 10.3389/fvets.2022.951176, 35990262 PMC9382074

[15] Vafa O Gilliland GL Brezski RJ Strake B Wilkinson T Lacy ER . An engineered fc variant of an IgG eliminates all immune effector functions via structural perturbations. Methods. (2014) 65:114–26. doi: 10.1016/j.ymeth.2013.06.035, 23872058

[16] Rodnina MV. Translation in prokaryotes. Cold Spring Harb Perspect Biol. (2018) 10:a032664. doi: 10.1101/cshperspect.a032664, 29661790 PMC6120702

[17] Ariyanti D Ikebukuro K Sode K. Artificial complementary chromatic acclimation gene expression system in *Escherichia coli*. Microb Cell Factories. (2021) 20:128. doi: 10.1186/s12934-021-01621-3, 34225717 PMC8256508

[18] Zhang J Du C Pan Y Zhang Z Feng R Ma M . Optimization of a novel expression system for recombinant protein production in CHO cells. Sci Rep. (2024) 14:24913. doi: 10.1038/s41598-024-76995-6, 39438721 PMC11496728

[19] Abdel-Motal UM Wang S Awad A Lu S Wigglesworth K Galili U. Increased immunogenicity of HIV-1 p24 and gp120 following immunization with gp120/p24 fusion protein vaccine expressing alpha-gal epitopes. Vaccine. (2010) 28:1758–65. doi: 10.1016/j.vaccine.2009.12.015, 20034607 PMC2866524

[20] Gribben JG Devereux S Thomas NS Keim M Jones HM Goldstone AH . Development of antibodies to unprotected glycosylation sites on recombinant human GM-CSF. Lancet. (1990) 335:434–7. doi: 10.1016/0140-6736(90)90665-r 1968169

[21] Runkel L Meier W Pepinsky RB Karpusas M Whitty A Kimball K . Structural and functional differences between glycosylated and non-glycosylated forms of human intrferon-beta (IFN-beta). Pharm Res. (1998) 15:641–9. doi: 10.1023/a:1011974512425 9587963

[22] Hayrapetyan H Tran T Tellez-Corrales E Madiraju C. Enzyme-linked immunosorbent assay: types and applications. Methods Mol Biol. (2023) 2612:1–17. doi: 10.1007/978-1-0716-2903-1_1, 36795355

[23] Jitsukawa T Nakajima S Sugawara I Mori S De Ley M. Characterization of murine monoclonal antibodies to human interferon-gamma (IFN-gamma) and their application for sandwich enzyme-linked immunosorbent assay (ELISA). Microbiol Immunol. (1987) 31:809–20. doi: 10.1111/j.1348-0421.1987.tb03142.x, 2446109

[24] Kim J-Y Castro F Shin S-M Kwon SR Jung S-J. Development of sandwich ELISA for IL-10 quantification in olive flounder (*Paralichthys olivaceus*) and its application to protein-level analysis during viral hemorrhagic septicemia virus (VHSV) infecton. Fish Shellfish Immunol. (2026) 173:111270. doi: 10.1016/j.fsi.2026.111270 41825729

[25] Tighe PJ Ryder RR Todd I Fairclough LC. ELISA in the multiplex era: potenials and pitfalls. Proteomics Clin Appl. (2015) 9:406–22. doi: 10.1002/prca.201400130, 25644123 PMC6680274

[26] Yin Z He Q Yang H Morisseau C El-Sheikh E-SA Li D . Development of a sandwich ELISA for the specific quantitation of hemagglutinin (HA)-tagged proteins during their inducible expression in *Escherichia coli*. Anal Bioanal Chem. (2023) 415:5563–74. doi: 10.1007/s00216-023-04846-w, 37505234 PMC10473979

[27] Foss S Sakya SA Aguinagalde L Lustig M Shaughnessy J Cruz AR . Human IgG fc-engineering for enhanced plasma half-life, mucosal distribution and killing of cancer cells and bacteria. Nat Commun. (2024) 15:2007. doi: 10.1038/s41467-024-46321-9, 38453922 PMC10920689

[28] Yang Y Song H Zhang K Wang S Zhao Y Zhang Q . Efficient eukaryotic expression and potent antiviral activity of a long-acting recombinant feline interferon-ω2-fc fusion protein against major feline viruses. Antivir Res. (2025) 243:106272. doi: 10.1016/j.antiviral.2025.106272, 40912602

[29] Chi H-J Park M Han J-K Kim S-M Kang S Yang J-H . APB-F1, a long-acting feline granulocyte colony-stimulating factor fusion protein, created by exploiting FL335, a chimeric fab specific for feline serum albumin. Vet Immunol Immunopathol. (2021) 240:110322. doi: 10.1016/j.vetimm.2021.110322 34509747

