# Supplementary material for: Development of a sandwich ELISA for recombinant feline GM-CSF and determination of its serum half-life
Source: Front Vet Sci. 2026 Oct 19;13:1958789. doi: 10.3389/fvets.2026.1958789 (PMC13725428; doi:10.3389/fvets.2026.1958789)
Supplement: Supplementary file 1 [file Table_1.DOCX]

**Supplementary Table S1. Individual serum concentration-time data of GM-CSF-His in cats after subcutaneous administration (10 μg/kg)**

| Time (h) | Cat 1 (pg/mL) | Cat 2 (pg/mL) | Cat 3 (pg/mL) |
| --- | --- | --- | --- |
| 1 | 46359.97 | 49228.75 | 44407.63 |
| 2 | 45178.27 | 52485.79 | 48526.60 |
| 4 | 36595.55 | 36890.28 | 35803.37 |
| 8 | 19603.59 | 16872.60 | 17578.99 |
| 16 | 3667.53 | 4063.64 | 4120.37 |
| 24 | 1226.23 | 1325.89 | 1312.66 |
| 32 | 467.12 | 366.24 | 394.16 |
| 48 | 78.60 | 84.60 | 97.20 |
| 72 | — | — | — |
| 96 | — | — | — |
| 120 | — | — | — |
| 144 | — | — | — |
| 168 | — | — | — |
| 192 | — | — | — |
| 216 | — | — | — |

Note: Data are presented as individual serum concentrations (pg/mL) for each cat in the GM-CSF-His group (n = 3). Cats were subcutaneously administered 10 μg/kg GM-CSF-His. Serum samples were collected at the indicated time points. “—” indicates that no sample was collected at that time point. Concentrations below the LLOQ (50 pg/mL) were reported as BLQ and are not shown in this table.

**Supplementary Table S2. Individual serum concentration-time data of GM-CSF-Fc in cats after subcutaneous administration (10 μg/kg)**

| Time (h) | Cat 1 (pg/mL) | Cat 2 (pg/mL) | Cat 3 (pg/mL) |
| --- | --- | --- | --- |
| 1 | 31200.29 | 27400.03 | 32600.50 |
| 2 | 33520.68 | 32650.57 | 35100.14 |
| 4 | 31120.52 | 30562.21 | 35982.65 |
| 8 | 29956.77 | 29012.05 | 33050.18 |
| 16 | 28642.95 | 27463.81 | 30975.92 |
| 24 | 25961.97 | 24764.12 | 27160.23 |
| 32 | 22630.30 | 21392.76 | 23630.76 |
| 48 | 18790.14 | 16300.48 | 18930.73 |
| 72 | 13564.00 | 11968.85 | 14360.25 |
| 96 | 8753.37 | 6932.11 | 9321.65 |
| 120 | 5512.37 | 5019.25 | 6040.96 |
| 144 | 3548.77 | 3216.97 | 3898.54 |
| 168 | 2846.73 | 2786.07 | 3064.39 |
| 192 | 1663.58 | 1422.07 | 1966.57 |
| 216 | 1026.01 | 878.27 | 1369.61 |

Note: Data are presented as individual serum concentrations (pg/mL) for each cat in the GM-CSF-Fc group (n = 3). Cats were subcutaneously administered 10 μg/kg GM-CSF-Fc. Serum samples were collected at the indicated time points. All concentrations remained above the LLOQ (50 pg/mL) throughout the sampling period.
